# Optimal composition of the poly(triarylamine)-based polymer composite to maximize photorefractive performance

**DOI:** 10.1038/s41598-018-36980-2

**Published:** 2019-01-24

**Authors:** Kento Masumura, Ikumi Nakanishi, Khanh Van Thi Khuat, Kenji Kinashi, Wataru Sakai, Naoto Tsutsumi

**Affiliations:** 1 Doctor’s Program of Materials Chemistry, Graduate School of Science and Technology, Kyoto Institute of Technology, Matsugasaki, Sakyo, Kyoto, 606-8585 Japan; 2 Master’s Program of Innovative Materials, Graduate School of Science and Technology, Kyoto Institute of Technology, Matsugasaki, Sakyo, Kyoto, 606-8585 Japan; 3 Faculty of Materials Science and Engineering, Kyoto Institute of Technology, Matsugasaki, Sakyo, Kyoto, 606-8585 Japan

## Abstract

A holographic display system requires the external diffraction efficiency to be greater than 10% and four orders of magnitude of sensitivity for practical usage. To achieve such requirements, the photorefractive (PR) performance of PR composite based on poly[bis(2,4,6-trimethylpheneyl)amine] (PTAA) has been investigated. In the present report, the change of the content of PTAA as a photoconductive polymer, (2,4,6-trimethylphenyl)diphenylamine (TAA) as a photoconductive plasticizer, and second trap agent bathophenanthroline (BPhen) reasonably optimized the PR response time and external diffraction efficiency. High sensitivity of 1851 cm^2^ J^−1^ with response time of 494 μs and external diffraction efficiency of 23.9% were achieved at 532 nm and 60 V μm^−1^ by reducing the content of PTAA and increasing the contents of TAA and BPhen. Decreasing the amount of PTAA and increasing the contents of TAA and BPhen lowered the absorption coefficient, resulting in the high external diffraction efficiency. The narrower distribution of the electronic density of states (DOS) for PTAA/TAA (43.5/20 and 33.5/30) also contributed to the shorter PR response time of hundreds of microseconds.

## Introduction

Photorefractive (PR) polymer composites are prospective candidates for use in holographic three-dimensional (3D) displays that provide realistic images without special eyeglasses^1–4^. PR polymer composites are superior to photopolymers in terms of image-updating capability and low cost^1^. PR polymer composites basically consist of photoconductive polymers, nonlinear optical materials, sensitizer, and plasticizer^5–10^. PR phenomena are based on the refractive index modulation through Pockels effect due to the space charge field induced by the redistribution of the photogenerated charge carriers^5–10^. The degree of modulation in refractive index determines the diffraction efficiency, and the speed of the redistribution of photogenerated charge carriers (the formation speed of the space charge field) determines the rise time of optical diffraction and amplification. Brightness of holographic images are related to the high optical diffraction and the smoothness of the dynamic holographic images by the short build up time of refractive index modulation. Time response of PR polymers is in the order of milliseconds or less than millisecond which corresponds to the video rate for holographic 3D display.

Photoconductive polymers (in general, p-type semiconductors) play an important role of the hole transport. The charges induced by the sensitizers are transported along the HOMO (highest occupied molecular orbital) region of the photoconductive polymers. NLO dyes contribute to enhance the refractive index modulation by reorientation under the Pockels effect. Plasticizers decrease *T*_g_ to improve the reorientation of the NLO dyes. N-type semiconductors are used sensitizers to generate charge carriers. Sensitizers need low LUMO (lowest unoccupied molecular orbital) to sensitize in the wavelength of writing beams. PR performance of PbS nanocrystals doped PR composite has been reported^11^. Diffraction response with wide range pulse duration has been investigated for photorefractive polymers^12^. In a series of research of PR composite based on poly[bis(2,4,6-trimethylpheneyl)amine] (PTAA) in our laboratory, the utilization of the self-assembled monolayer (SAM) between PR composite and indium tin oxide (ITO) electrode drastically reduced the extremely large photocurrent^13^, which contributes to the significant improvement of the diffraction response in the order of milliseconds^14^. The addition of the second electron trap reagent effectively reduce the photo current to achieve the time response in diffraction in the order of sub-milliseconds^15,16^; the addition of tris-(8-hydroxyquinoline)aluminum (Alq_3_) leads to the PR response of 860 μs^15^, and the addition of bathophenanthroline (BPhen) leads to 397 μs response time^16^, which can be compared with the recent PR sub-millisecond response time of 399 μs^11^. However, despite the higher internal diffraction efficiency above 70% was measured, a low external diffraction efficiency below 6% was observed because of the higher absorption coefficient of the PR composites based on PTAA^16^. To achieve the external diffraction efficiency greater than 10%, further optimization of each composition for the PR composites based on PTAA is required to decrease the absorption coefficient. Above all, higher external diffraction efficiency will directly improve the sensitivity of PR composites.

In the present report, we optimized the ratio of the PTAA, TAA and BPhen to obtain the enhanced PR performance and achieved the higher external diffraction efficiency of 23.9% and higher sensitivity of 1851 cm^2^ J^−1^ at 532 nm and 60 V μm^−1^ for the PR composite of PTAA/PDCST/TAA/PCBM/BPhen with weight ratio of 31.5/35/30/0.5/3. The low absorption coefficient by the optimization of the composite is the key point for higher external diffraction efficiency and thus higher sensitivity. The empirical sensitivity, containing the elements of the response time and the external diffraction efficiency, was estimated to indicate the most promising candidate of the PR composite based on PTAA as real-time 3D holographic displays.

## Experiments

### Preparation of materials and composites

**Figure 1:**
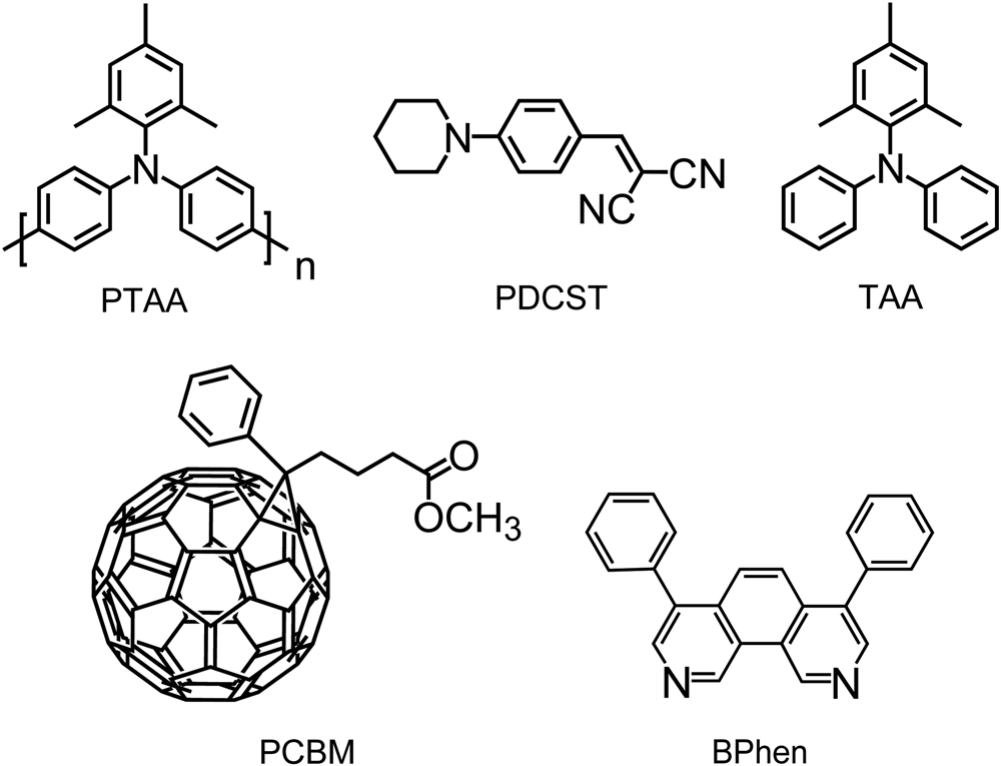
Structural formulae of the materials.

Materials whose structural formulae are shown in Fig. 1 are used in this study. PTAA was purchased from Sigma-Aldrich Co., USA to use as a photoconductive polymer. PTAA was reprecipitated by dropping PTAA chloroform solution into the excess amount of hexane. Optical nonlinear dye of piperidinodicyanostyrene (PDCST) and photoconductive plasticizer of (2,4,6-trimethylphenyl)diphenylamine (TAA) synthesized in our laboratory were used^14^. Phenyl-C_61_-butyric acid methyl ester (PCBM) as a sensitizer and BPhen as a second electron trap were purchased from Tokyo Kasei Co., Japan. PCBM and BPhen were used as received.

PDCST, PCBM and BPhen were mixed with a fixed ratio of 35, 0.5 and 1 by weight, respectively. The composition ratio of PTAA and TAA were changed to 53.5/10, 43.5/20, 33.5/30 and 23.5/40 by weight. PTAA, PDCST, TAA, PCBM, and BPhen were mixed in a tetrahydrofuran (THF) with stirring for 24 h. Then PR solution was cast on a stage heated at 70 °C for 24 h to obtain PR composite. The same procedure described in previous report^16^ was used: prior to the preparation of ITO/sample/ITO device, ITO electrodes were modified with aminopropyltrimethoxysilane (APTMS) to form SAM on a stage heated at 160 °C. Then the samples were sandwiched between two SAM modified ITO electrodes. Teflon spacer was used to adjust the thickness of PR composite film to 50 μm. The more detailed SAM-ITO preparation method is presented in a previous report^13^.

### Measurements

Absorption spectrum of each PR composite in the UV-Vis region is recorded on a Ultraviolet/Visible/Near infrared spectrophotometer (Lambda1050, Perkin-Elmer Co., USA). The absorption coefficient *α* is evaluated using *α* = *A*ln(10)/*d* with the absorbance *A* and the thickness *d* of the PR composite film.

A degenerate four-wave mixing (DFWM) technique is used to evaluate the efficiency for optical diffraction and its response time of the PR composite. *s*-Polarized beams of a are intersected in the PR composite to write PR gratings. The incidence angle of two intersecting beams normal to the PR device is 42.5° and 57.5°. The corresponding internal angles of *θ*_A_ = 23.42° and *θ*_B_ = 29.74° are calculated with an index of refraction, *n* = 1.7. Laser source is a DPSS laser (25 mW, @ 532 nm, Cobolt AB, Sweden). Laser power per unit area is 0.534 W cm^−2^. To read the gratings a *p*-polarized probe beam with much weaker intensity from the same laser source is oppositely propagated through PR device. Grating information is evaluated from the diffracted signal of probe beam. A fast high-voltage amplifier (Trek model 10/10E, USA) is used to supply a rectangular high voltage at 100 Hz to the PR composites. Two photodiode detectors are used to detect the diffracted and transmitted signals. The internal diffraction efficiency (*η*) is evaluated using the Eq. (1):

1 $$\eta ( \% )=\frac{{I}_{{\rm{d}}}}{{I}_{{\rm{t}}}+{I}_{{\rm{d}}}}\times 100$$

where *I*_d_ is the intensity of the diffracted beam and *I*_t_ is that of the transmitted beam. To estimate the response time (*τ*) of the optical diffraction, a time trace of *η* is fitted by a Kohlrausch-Williams-Watts (KWW) stretched exponential function of Eq. (2):

2 $$\eta ( \% )={\eta }_{0}\{1-\exp [-{(\frac{t}{\tau })}^{{\rm{\beta }}}]\}$$

where *η*_0_ is the diffraction efficiency at the steady-state condition, *t* is the time, and *β* (0 < *β* ≤ 1) is a dispersion parameter deviated from a single exponential fitting related to the release time from the traps.

The external diffraction efficiency *η*_ext_ is evaluated using Eq. (3) with the internal diffraction efficiency *η*, absorption coefficient *α*, thickness of sample device *d,* and the internal angle of beam A *θ*_A_:

3 $${\eta }_{{\rm{ext}}}=\exp (-\frac{\alpha d}{\cos \,{\theta }_{{\rm{A}}}})\eta $$

The empirical sensitivity *S* is calculated using Eq. (4) with the external diffraction efficiency *η*_ext_, the response time *τ*, and the laser intensity *I* (in the present case, *I* = 0.534 Wcm^−2^):

4 $$S=\frac{\sqrt{{\eta }_{{\rm{ext}}}}}{I\tau }$$

A current flow on the light illumination (photocurrent) was recorded by a current monitor in a Trek 610E high-voltage amplifier when a DFWM signal was measured.

## Results and Discussion

### Optimization of the plasticizer content for PR composite based on PTAA

**Figure 2:**
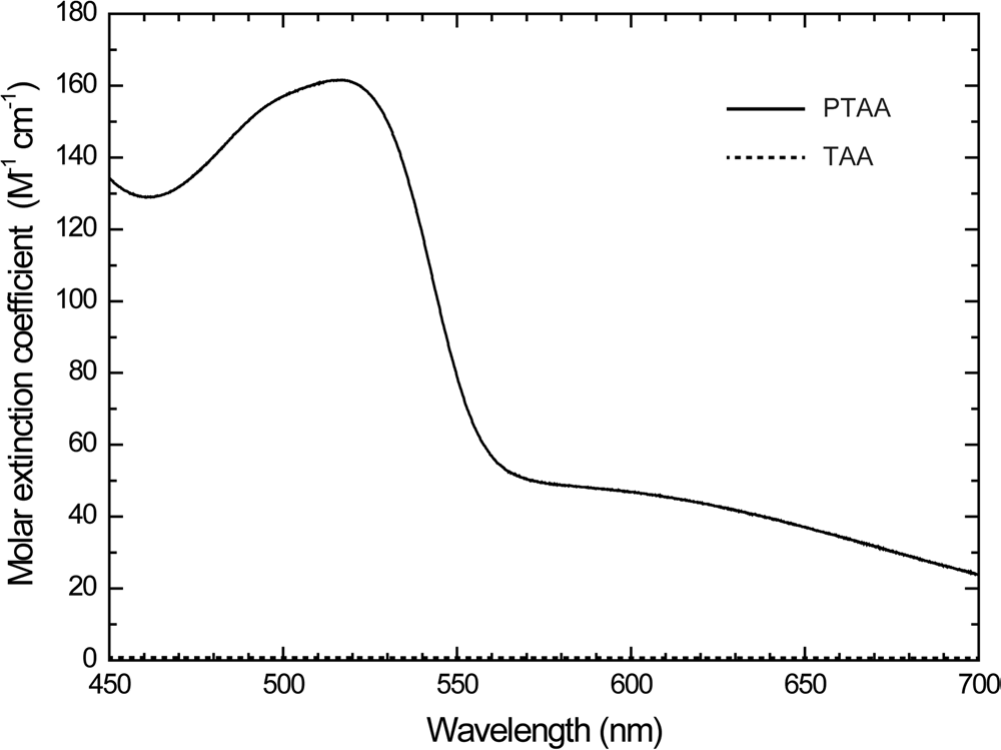
UV-Vis absorption spectrum of the PTAA and TAA in THF solution.

A photorefractive rise time of 397 μs and a high diffraction efficiency of 73% were successfully achieved by inducing a second electron trap BPhen in PR composite based on PTAA^16^. However, the low external diffraction efficiency of 6% was caused by the high absorption coefficient of the composite. The external diffraction efficiency is an important parameter for evaluating the device performance. As shown in equation (3), the external diffraction efficiency is significantly influenced by the absorption coefficient at the reading wavelength of the probe beam. The UV-Vis spectrum of the PTAA and TAA in THF solution is shown in Fig. 2. TAA is a monomeric unit of PTAA and functions as a photoconductive plasticizer in the PR composites. PTAA has a broad absorption spectrum below 550 nm, whereas TAA does not have any absorption in the visible region. The wavelength of the writing beam is 532 nm^16^. Therefore, the content of PTAA significantly influences the absorption coefficient of the PR composites based on PTAA.

**Figure 3:**
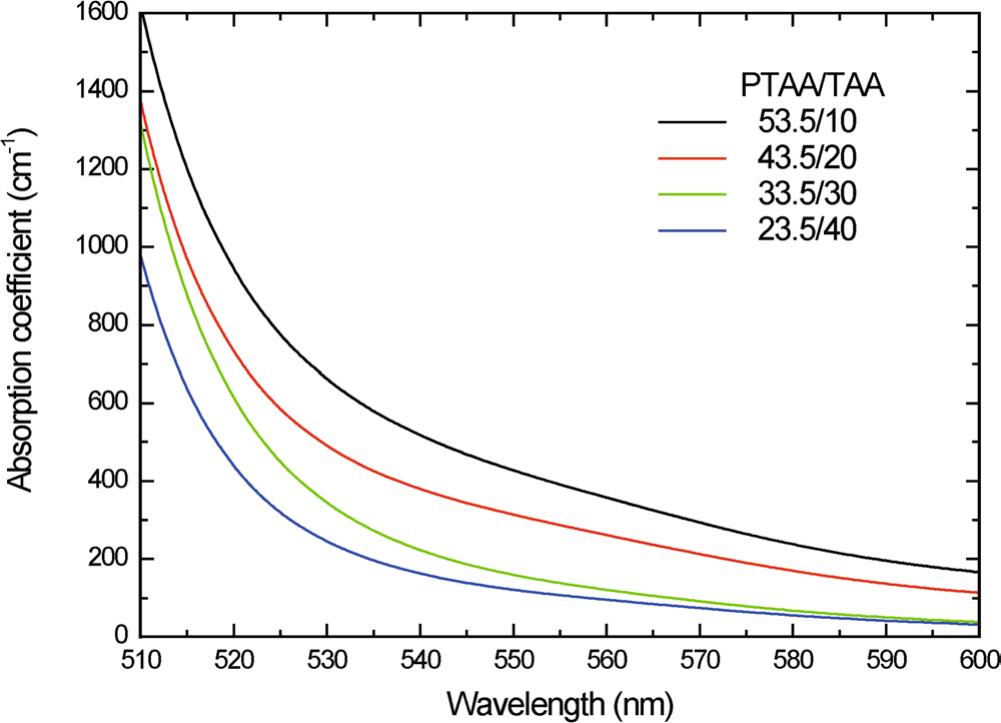
UV-Vis absorption spectra of PR composites based on PTAA.

**Figure 4:**
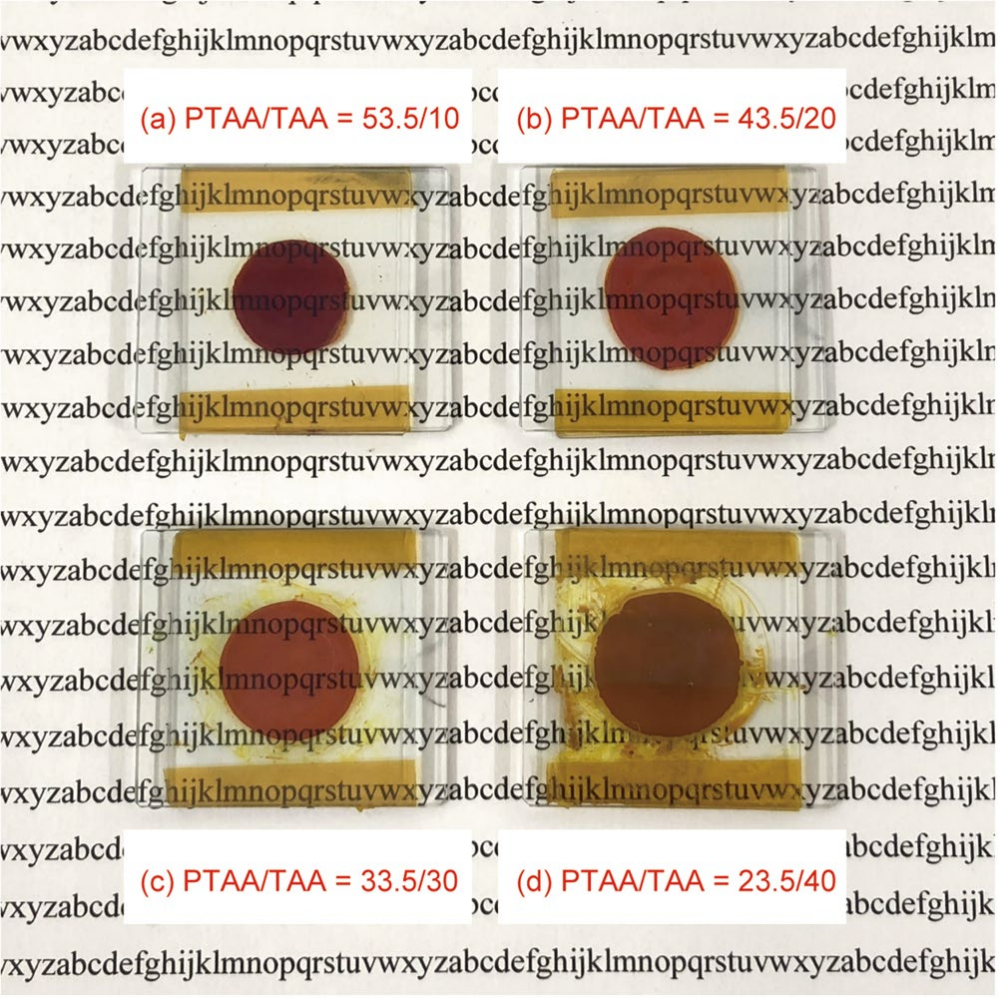
Photograph of PTAA-based PR composites after one day of storage in the room temperature of 23 °C: (**a**) PTAA/TAA = 53.5/10, (**b**) PTAA/TAA = 43.5/20, (**c**) PTAA/TAA = 33.5/30, and (**d**) PTAA/TAA = 23.5/40.

The UV-Vis absorption spectra of the composites of PTAA/TAA (63.5-X/X) are shown in Fig. 3. With reduced content of PTAA (with increasing content of TAA), the absorption coefficient at 532 nm was largely decreased. Figure 4 shows the photograph of each composite after one day of storage in the room temperature of 23 °C. As can be seen in Fig. 4(a–c), the PTAA-based composites show enough clear to see the letters behind them. On the other hand, the PTAA/TAA (23.5/40) sample in Fig. 4(d) becomes opaque because of a phase separation caused by dye aggregation in the sample after one day of storage. The main cause of dye aggregation was the low content of polymer component in the composite.

**Figure 5:**
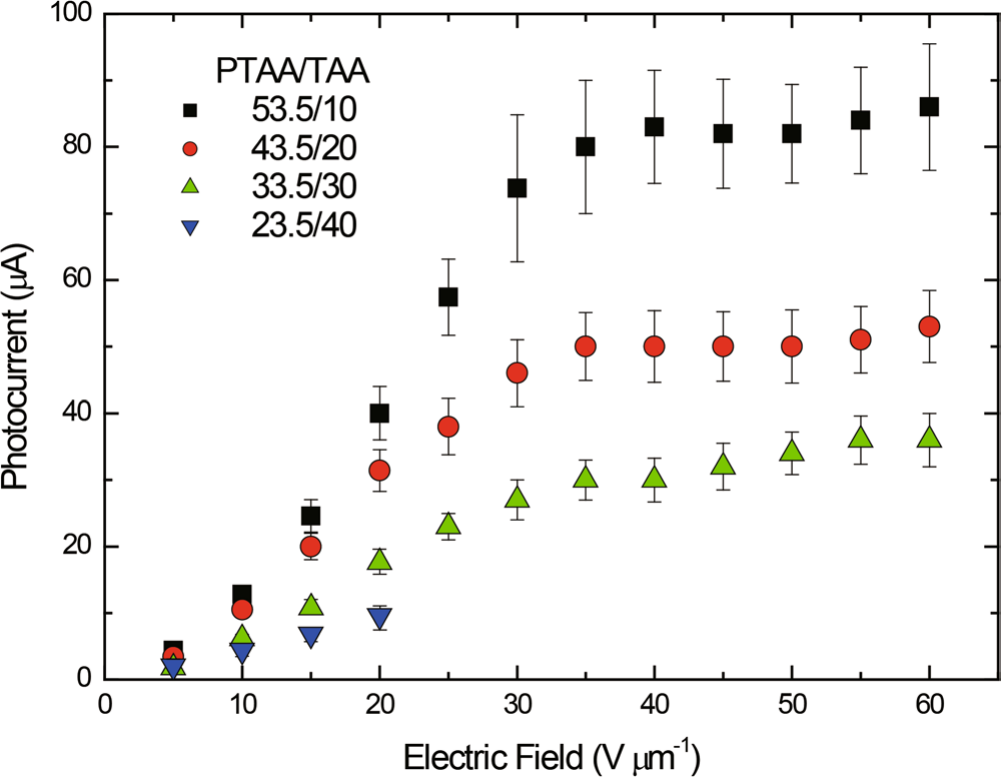
Plots of photocurrent as a function of applied field for PTAA PR composites of PTAA/PDCST/TAA/PCBM/BPhen (53.5/35/10/0.5/1: squares, 43.5/35/20/0.5/1: circles, 33.5/35/30/0.5/1: triangles, 23.5/35/40/0.5/1: inverted triangles).

The plots of photocurrent with the electric field is shown in Fig. 5. The photocurrent is found to decrease with decreasing the content of PTAA. Dielectric breakdown occurred at 25 V μm^−1^ via the dye aggregation in the PTAA/TAA (23.5/40) PR composite.

Diffraction responses are significantly affected by the space charge formed under an intersected optical illumination with applied electric field. To understand the optical diffraction dynamics, the response time profile of the diffraction is investigated as the light or the electric field is turned on. In former report^15^, we measured the response times by using both methods. Generally, the former method was considered to be suitable, however, within 20% difference of the response times was found.

**Figure 6:**
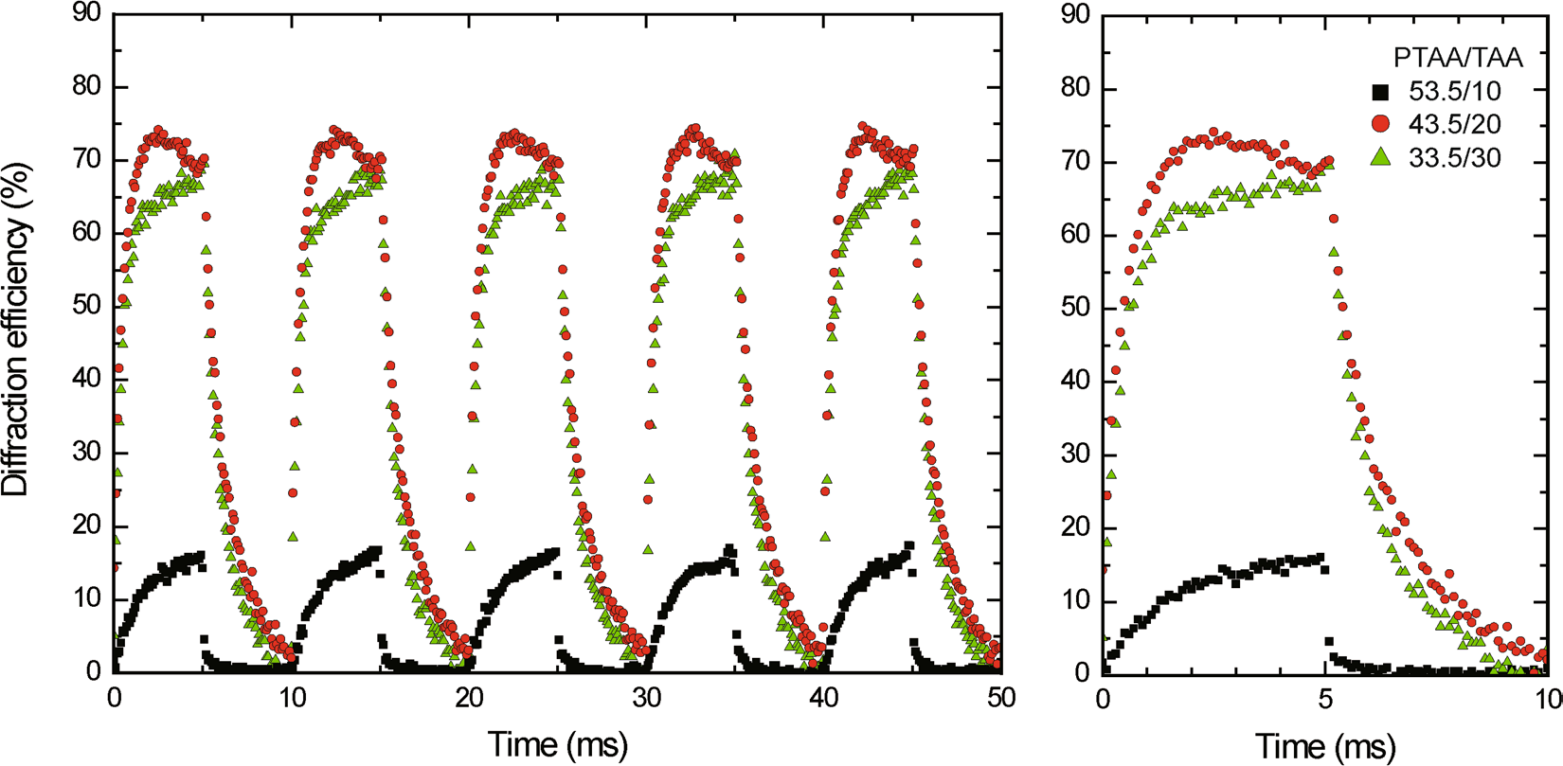
Left: continuous measurement of diffraction efficiency by turning on and off rectangular applied electric field (from 0 to 60 V μm^−1^) at 100 Hz for the PR composites of PTAA/PDCST/TAA/PCBM/BPhen (solid squares: 53.5/35/10/0.5/1, solid circles: 43.5/35/20/0.5/1, solid triangles: 33.5/35/30/0.5/1 by wt.). Right: Extracted profile for one cycle response.

**Figure 7:**
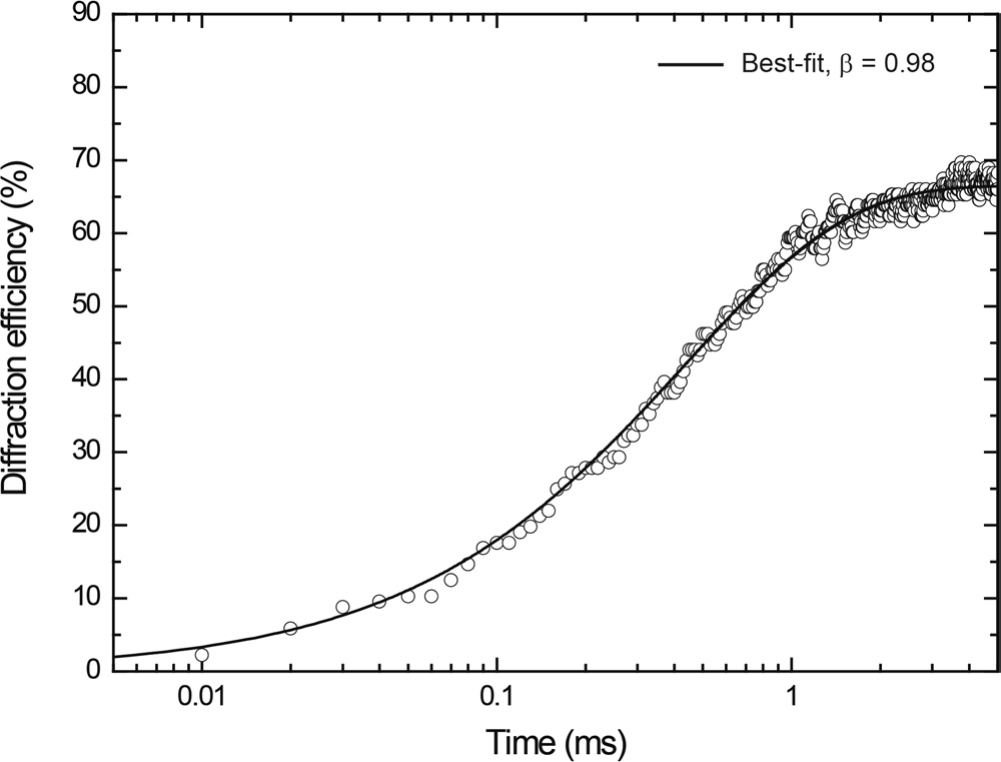
The build up time profile of the optical diffraction with time in the logarithmic scale for the PR composite of PTAA/PDCST/TAA/PCBM/BPhen with weight ratio of 33.5/35/30/0.5/1. Solid curve is the fitted KWW plots with *τ* of 422 μs and *β* = 0.98.

Then, the response dynamics were evaluated from the continuous measurements of the diffraction efficiency by turning on and off the rectangular electric field (from 0 to 60 V μm^−1^) at 100 Hz. The plots of the diffraction efficiencies with time are shown for the PR composites with changing the PTAA/TAA ratio between 53.5/10, 43.5/20, and 33.5/30 by wt in Fig. 6. Other weight ratio of PDCST/PCBM/BPhen is fixed 35/0.5/1. The rise and decay of the diffraction efficiency by turning on and off the applied rectangular field are directly related to the presence and absence of space charge field, respectively. Figure 7 shows the build up time profile of the optical diffraction with time in the logarithmic scale. The obtained optical diffraction curve from the building up region to the saturation region is fitted well by a stretched exponential function of KWW with *τ* of 422 μs and *β* = 0.98.

The PR quantities and absorption coefficients of the PR composites based on PTAA are summarized in Table 1. The diffraction efficiencies were 16.8, 73.3, and 66.5% for the PTAA/TAA (53.5/10), PTAA/TAA (43.5/20), and PTAA/TAA (33.5/30), respectively. The PR response times were measured to be 1578, 397 and 422 μs for the PTAA/TAA (53.5/10), PTAA/TAA (43.5/20), and PTAA/TAA (33.5/30), respectively. The diffraction efficiency and response time of the PTAA/TAA (43.5/20) are comparable to those for the PTAA/TAA (33.5/30). However, the external diffraction efficiency of the PTAA/TAA (33.5/30) was measured to be 12.1%, which is twice that of PTAA/TAA (43.5/20). This high external diffraction efficiency is caused by the low absorption coefficient of the PTAA/TAA (33.5/30). The resulting sensitivity, including the parameters of external diffraction efficiency and response time, was calculated to be 1541 cm^2^ J^−1^ for PTAA/TAA (33.5/30). The short response time and the high external diffraction efficiency resulted in the high sensitivity.

**Table 1:** PR quantities and absorption coefficient for the PR composites based on PTAA with various contents of TAA.

PTAA/TAA (wt%)	*α* (cm^−1^)	*Δn*	*Γ* (cm^−1^)	*η* /*η*_ext_ (%)	*τ* (μs)/*β*	*S* (cm^2^ J^−1^)
53.5/10	625	1.28 × 10^−3^	63	16.8/0.6	1578/0.93	89
43.5/20	462	3.13 × 10^−3^	156	73.3/5.9	397/0.54	1145
33.5/30	313	2.90 × 10^−3^	215	66.5/12.1	422/0.98	1541
23.5/40	223			—*		

*Dielectric breakdown below 25 V μm^−1^.

### Photorefractive response time

In previous report, we have discussed that the photorefractive response time can be predicted from the photoconductivity using the relation of

5 $${\tau }_{{\rm{G}}}=\frac{{\varepsilon }_{{\rm{r}}}{\varepsilon }_{0}}{{\sigma }_{{\rm{ph}}}}$$

where *τ*_G_ is the build-up time to establish the steady-state space-charge field^15,16^. Here *ε*_r_ is a dielectric constant, and *ε*_0_ is permeability in vacuum. The trap density *T*_i_ at initial stage in Schildkraut’s model^17^ is related to the internal photocurrent efficiency *φ*_ph_ (*E*) as

6 $${\phi }_{{\rm{ph}}}(E)=G{\eta }_{{\rm{p}}}$$

where *G* is the gain factor for photoconductivity, $G=\frac{{\varepsilon }_{{\rm{r}}}{\varepsilon }_{0}{E}_{0}}{eL{T}_{{\rm{i}}}}$^15,18^. Here *E*_0_ is the applied electric field, *e* is the elementary charge, and *L* is the thickness of the PR sample. *φ*_ph_ (*E*) is also defined as ${\phi }_{{\rm{ph}}}(E)=\frac{{J}_{{\rm{ph}}}h\nu }{e{I}_{0}\alpha L}=\frac{{\sigma }_{{\rm{ph}}}{E}_{0}h\nu }{e{I}_{0}\alpha L}\,$, where *J*_ph_ is the photocurrent per unit area, *σ*_ph_ is the photoconductivity, *h* is the Planck constant, *ν* is the optical frequency of the illuminated light, *I*_0_ is the light intensity, and *α* is the absorption coefficient^15,16,18,19^. The detailed procedures were described in previous reports^15,16^.

Table 2 summarizes the value of *φ*_ph_ calculated. With the *ε*_r_ of 3.5 and the *T*_i_ value of 8.00 × 10^14^ cm^−3^ ^16^, *η*_p_ is estimated for the PR composite of PTAA/TAA (43.5/20). Assuming that *η*_p_ is proportional to *I*_p_ (*J*_ph_) and doesn’t not exceed a unity, the trap densities of *T*_i_ are estimated at 9.48 × 10^14^ cm^−3^ for PTAA/TAA (53.5/10) and 5.42 × 10^14^ cm^−3^ for PTAA/TAA (33.5/30). The calculated *η*_p_, *G*, *T*_i_, *E*_q_, and *τ*_G_ are summarized for each sample in Table 2.

**Table 2:** Parameters related to the photocurrent and PR properties in the PR composites based on PTAA with various contents of TAA.

PTAA/TAA (wt%)	*E*_0_ (V μm^−1^)	*I*_p_ (μA)	*J*_ph_ (μA cm^−2^)	*σ*_ph_ (nS cm^−1^)	*φ*_ph_	*η*_p_	*G*	*T*_i_ (cm^−3^)	*E*_q_ (V μm^−1^)	*τ* (μs)	*τ*_G_ (μs)	*N*_c_ (s^−1^ cm^−3^)
53.5/10	60	86	3927	6.54	0.0063	0.0256	0.245	9.48 × 10^14^	2.3	1578	47	6.0 × 10^17^
43.5/20	60	53	2420	4.03	0.0046	0.0158	0.290	8.00 × 10^14^	1.9	397	77	2.0 × 10^18^
33.5/30	60	36	1644	2.74	0.0046	0.0107	0.428	5.42 × 10^14^	1.3	422	113	1.3 × 10^18^

Here, the measured response *τ* is much slower than *τ*_G_. For the estimation of *τ*_G_, all charge carriers optically generated contribute to fill the traps ${\tau }_{{\rm{G}}}=\frac{{T}_{{\rm{i}}}}{(\frac{\alpha {\eta }_{{\rm{p}}}{I}_{0}}{h\nu })}$ ^20^. However, for the present PR polymer composites, this assumption is rarely valid; thus, we introduce the reasonable relation of

7 $$\tau =\frac{{T}_{{\rm{i}}}}{{N}_{{\rm{c}}}}$$

in previous report^16^. Here, *N*_C_ is the total number of charge carriers trapped per unit volume. Evaluation of *N*_C_ for three PTAA-based PR composites is as follows; *Ν*_C_ (PTAA/TAA (53.5/10)) < *Ν*_C_ (PTAA/TAA (33.5/30)) < *Ν*_C_ (PTAA/TAA (43.5/20)). The present evaluation of *N*_C_ means that higher PTAA content leads to lower trap density, which will contradict the common understanding for trap density in polymers. The trap density in polymers is commonly understood to be higher than that for a low molecular weight monomeric unit compound, i.e., the trap density of the PR composite with higher content of PTAA will be higher. However, the present results show that PTAA/TAA (53.5/10) has lower *N*_C_. Thus, another reasonable explanation should be considered.

**Figure 8:**
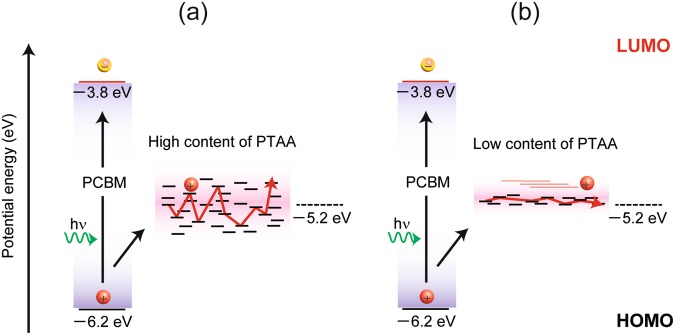
Hole-hopping mechanism of PTAA-based PR composite: (**a**) composite with high content of PTAA; (**b**) composite with low content of PTAA. The CT complex between PTAA and BPhen is omitted for simplification.

In the present case, a total number of hopping sites of TAA monomeric units per unit volume in the PR composite of PTAA/TAA is constant at 1.6 × 10^21^ cm^−3^ for three PR composites of PTAA/TAA (53.5/10, 43.5/20, 33.5/30). On one hand, the total number of hopping sites is calculated using composite density, molecular weight of TAA, total weight percentages of PTAA and TAA, and Avogadro’s number. On the other hand, the energy distribution of each hopping site will be significantly affected by the numbers of the components of TAA and PTAA. The energy distribution of hopping sites in polymer is commonly wide compared to the corresponding monomeric low molecular weight compound. Thus, the electronic density of state (DOS) of hopping sites becomes wider for the polymer-rich PR composite. In a former study by our laboratory^21^, narrower DOS led to the shorter response time, whereas wider DOS led to the longer response time for the PR composite of poly(4-(diphenylamino)benzyl acrylate) (PDAA) and (4-(diphenylamino)phenyl)methanol (TPAOH). PDAA is photoconductive polymer, and TPAOH is a monomeric unit of PDAA that functions as a photoconductive plasticizer, i.e., the decrease of PDAA content led to narrower DOS bandwidth. The narrower DOS distribution results in the faster charge transport and shorter response time. The similarity between PTAA/TAA and PDAA/TPAOH assumes that the same phenomenon occurs in the PR composite based on PTAA/TAA. The predicted energy distribution of hopping sites for the PR composite based on PTAA/TAA is shown in Fig. 8. A higher content of PTAA in the case of PTAA/TAA (53.5/10) results in a wider distribution of DOS, leading to the slower response of diffraction. In contrast, the lower content of PTAA in the case of PTAA/TAA (43.5/20) and PTAA/TAA (33.5/30) gives a narrower distribution of DOS, leading to the faster response of diffraction. The PR diffraction response of PTAA/TAA (43.5/20) and PTAA/TAA (33.5/30) was nearly equivalent; thus, 20 wt% of TAA is sufficient for forming a narrower distribution of DOS in PTAA/TAA PR composite.

### Optimizing the content of BPhen

For further optimization of the PR composites based on PTAA/TAA, the effect of the BPhen content on PR performances is investigated. In former report^16^ on PR composites based on PTAA, by inducing BPhen as the second electron trap, a sub-millisecond PR response with decreasing photocurrent was achieved. By adding a higher content of BPhen and decreasing the content of PTAA, lower absorption coefficient and photocurrent is expected, leading to higher external diffraction efficiency.

**Figure 9:**
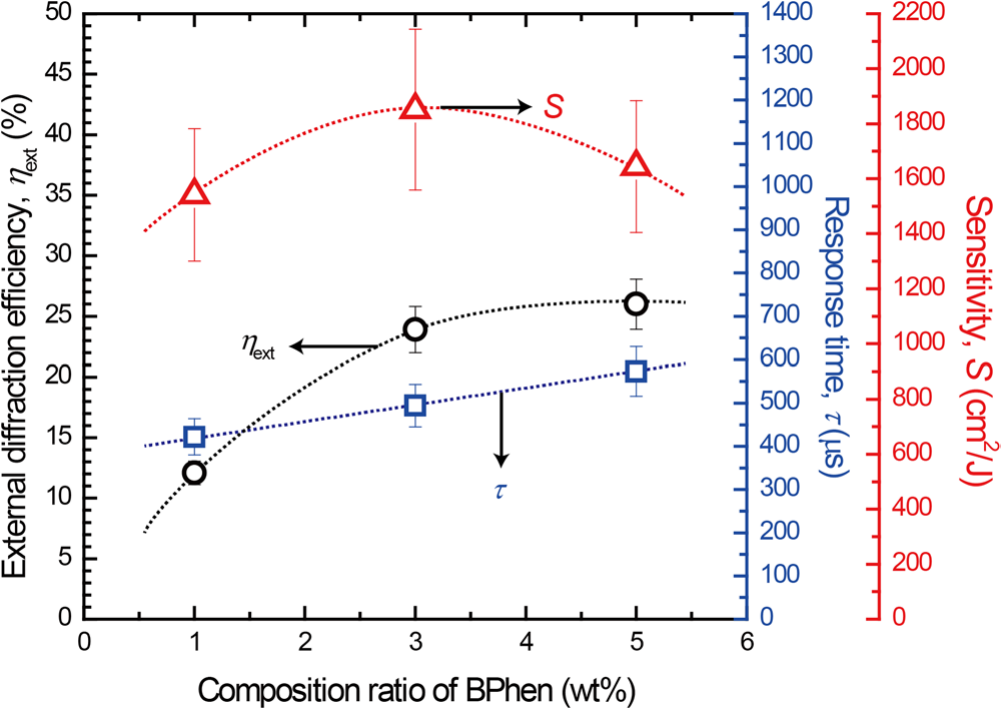
Plots of external diffraction efficiency, response time, and sensitivity as a function of BPhen content under a rectangular applied field at a frequency of 100 Hz (from 0 to 60 V μm^−1^).

The PR parameters and related quantities of the composites are summarized when the content of BPhen is changed from 1 to 5 wt% in Table 3. Increasing the content of BPhen, the absorption coefficient and the photocurrent of PR composites are decreased. The obtained results of *η*_ext_, *τ* and *S* are plotted as a function of BPhen content in Fig. 9. *η*_ext_ is increased with increasing content of BPhen, and longer *τ* was measured. A maximum sensitivity of 1851 cm^2^ J^−1^ is calculated for the PR composite of PTAA/PDCST/TAA/PCBM/BPhen with weight ratio of 31.5/35/30/0.5/3.

**Table 3:** PR parameters and absorption coefficient and photocurrent in the PR composites based on PTAA with various contents of BPhen.

BPhen content (wt%)	*α* (cm^−1^)	*I*_p_ (μA)	*Δn*	*η* (*η*_ext_) (%)	*τ* (μs)/*β*	*S* (cm^2^ J^−1^)
1	313	36	2.90 × 10^−3^	66.5 (12.1)	422/0.98	1541
3	205	22	3.12 × 10^−3^	73.1 (23.9)	494/0.98	1851
5	191	14	3.14 × 10^−3^	73.7 (26.0)	573/0.98	1664

## Conclusion

We have investigated the effects of the composition of each component of PTAA, PDCST, TAA, PCBM, and BPhen on the PR performance, especially the external diffraction efficiency and the sensitivity. By optimizing the contents of TAA and BPhen, higher PR performances were achieved: response time of 494 μs, external diffraction efficiency of 23.9%, and sensitivity of 1851 cm^2^ J^−1^ at 60 V μm^−1^ for the PR composite of PTAA/PDCST/TAA/PCBM/BPhen with weight ratio of 31.5/35/30/0.5/3. The high external diffraction efficiency was caused by the reduction of the absorption coefficient of the PR composite based on PTAA by decreasing the contents of PTAA and increasing the contents of TAA and BPhen. The small content of PTAA also contributed to the short response time by forming a narrower DOS distribution.

## References

[1] Tay S (2008). An updatable holographic three-dimensional display. Nature.

[2] Blanche P (2008). An updatable holographic display for 3D visualization. J. Display Tech.

[3] Blanche P–A (2010). Holographic three-dimensional telepresence using large-area photorefractive polymer. Nature.

[4] Lynn B, Blanche P–A, Peyghambarian N (2014). Photorefractive polymers for holography. J. Polym. Sci., B, Polym. Phys..

[5] Moerner WE, Scott M (1994). Silence, Polymeric photorefractive materials. Chem. Rev..

[6] Ostroverkhova O, Moerner WE (2004). Organic photorefractive: mechanisms, materials, and applications. Chem. Rev..

[7] Thomas J, Norwood RA, Peyghambarian N (2009). Non-linear optical polymers for photorefractive applications. J. Mater. Chem..

[8] Tsutsumi N (2016). Molecular design of photorefractive polymers. Polym. J..

[9] Tsutsumi, N. & Kenji, K. Photorefractive response: an approach from the photoconductive properties. In *Photorefractive Organic Materials and Applications* ed. Pierre-Alexandre Blanche, Springer, Chap. 3 (2016).

[10] Tsutsumi N (2017). Recent advances in photorefractive and photoactive polymers for holographic applications. Polym. Int..

[11] Moon J–S (2016). Sub-millisecond response time in a photorefractive composite operating under CW conditions. Sci. Rep..

[12] Blanche P–A (2016). Diffraction response of photorefractive polymers over nine orders of magnitude of pulse duration. Sci. Rep..

[13] Kinashi K, Shinkai H, Sakai W, Tsutsumi N (2013). Photorefractive device using self-assembled monolayer coated indium-tin-oxide electrodes. Org. Electron..

[14] Tsutsumi N, Kinashi K, Masumura K, Kono K (2015). Photorefractive performance of poly(triarylamine)-based polymer composites: An approach from the photoconductive properties. J. Polym. Sci., B, Polym. Phys..

[15] Tsutsumi N, Kinashi K, Masumura K, Kono K (2015). Photorefractive dynamics in poly(triarylamine)-based polymer composites. Opt. Express.

[16] Masumura K, Oka T, Kinashi K, Tsutsumi N (2018). Photorefractive dynamics in poly(triarylamine)-based polymer composite: An approach utilizing a second electron trap to reduce the photoconductivity. Opt. Material Express.

[17] Schildkraut JS, Buettner AV (1992). Theory and simulation of the formation and erasure of space charge gratings in photoconductive polymers. J. Appl. Phys..

[18] Däubler TK, Bittner R, Meerholz K, Cimrová V, Neher D (2000). Charge carrier photogeneration, trapping, and space-charge field formation in PVK-based photorefractive materials. Phys. Rev. B.

[19] Chantharasupawong P (2014). Photorefractive performances of a graphene-doped PATPD/7-DCST/ECZ composite. J. Mater. Chem. C.

[20] Däubler TK (2002). Photoconductivity and charge-carrier photogeneration in photorefractive polymers. Proc. SPIE.

[21] Nguyen TV, Kinashi K, Sakai W, Tsutsumi N (2016). Enhanced photorefractivity of a perylene bisimide-sensitized poly(4-(diphenylamino)benzyl acrylate) composite. Opt. Material Express.

